# The nutritional profile, mineral content and heavy metal uptake of yellow mealworm reared with supplementation of agricultural sidestreams

**DOI:** 10.1038/s41598-023-38747-w

**Published:** 2023-07-18

**Authors:** Isabelle Noyens, Floris Schoeters, Meggie Van Peer, Siebe Berrens, Sarah Goossens, Sabine Van Miert

**Affiliations:** RADIUS, Thomas More University of Applied Sciences, Kleinhoefstraat 4, 2440 Geel, Belgium

**Keywords:** Environmental impact, Entomology

## Abstract

Insect farming, a potential approach to deal with the increasing global protein demand, is a new activity in the Western world with many unanswered questions regarding product quality and safety. Insects may fulfill an important role in a circular economy by upcycling biowaste into valuable biomass. About half of the total mass of mealworm feeding substrates exists out of wet feed. This can be sourced from biowaste, increasing the sustainability of insect farming. This paper reports on the nutritional profile of yellow mealworm, *Tenebrio molitor*, reared with supplementation of organic sidestreams. These included unsold vegetables, potato cuttings, fermented chicory roots and horticultural foliage. The evaluation was performed by analyzing proximate compositions, fatty acid profiles, mineral and heavy metal contents. Mealworms fed with potato cuttings doubled their fat content and increased saturated and mono-unsaturated fatty acids. Providing fermented chicory roots increased the mineral content and accumulated heavy metals. Additionally, the uptake of minerals by mealworms was selective as only calcium, iron and manganese concentrations increased. Adding vegetable mix or horticultural foliage to the diet did not significantly change the nutritional profile. In conclusion, sidestreams were successfully recycled into protein-rich biomass and their nutrient content and bio-availability influenced the composition of mealworms.

## Introduction

The continuously growing human population is estimated to reach 9.7 billion people by 2050^1,2^, putting pressure on our food production to cope with the high food demand. This food demand is estimated to increase by 70–80% between 2012 and 2050^3–5^. Natural resources in our current food production are depleting, threatening our ecosystems and food supplies. Additionally, excessive amounts of biomass related to the production and consumption of food are wasted. Annual global waste is expected to reach 27 billion tons by 2050 of which a large fraction is biowaste^6–8^. In light of these challenges, innovative solutions, food alternatives and sustainable development of agricultural and food systems are proposed^9–11^. One such approach is the use of organic residual streams to generate feedstock, like the use of edible insects as a sustainable food and feed source^12,13^. Insect rearing produces less greenhouse gas and ammonia emissions, requires less water than conventional protein sources and can be produced in vertical farming systems, leading to a lower requirement of space^14–19^. It has been shown that insects are able to convert low-value biowaste into valuable protein-rich, up to 70% of the dry matter, biomass^20–22^. Furthermore, low-value biomass is now used for energy production, land filling or is disposed of and therefore does not compete with the current food and feed sector^23–26^. Considered as one of the most promising species for mass production for food and feed applications is the yellow mealworm, *Tenebrio molitor (T. molitor)*^27^. Both the larval and adult stage feed on a wide range of materials such as cereal products but also animal waste materials, vegetables, fruits, etc.^28,29^. *T. molitor* has been artificially reared on a small scale in Western societies, mostly as feed for hobby animals (e.g., birds or reptiles). Currently, their potential in food and feed applications has gained more attention^30–32^. For example, a novel food dossier was approved for *T. molitor* including applications, namely in frozen, dried and powder form (Regulation (EC) No 258/97 and Regulation (EU) 2015/2283)^33^. However, the mass production of insects for food and feed applications is still a relatively new concept in Western countries. This industry is facing some challenges such as knowledge gaps on optimal diets and production, nutritional quality of their end products and safety concerns such as toxicant accumulations and microbial hazards. Contrary to traditional livestock, the cultivation of insects lacks the same historical experience^17,24,25,34^.

Despite many studies were done on the nutritional composition of *T. molitor,* factors influencing their nutritional value are still not fully understood. Previous research indicates that the insect’s diet can be reflected in their composition to some extent, but no clear patterns have yet been identified. Furthermore, these studies focus on the protein and fat profiles of *T. molitor*, but the influence on the mineral profiles is limited^21,22,32,35–40^. More research is needed to understand the capacity of mineral uptake. A recent study concluded that the concentration of some minerals slightly increased in mealworm larvae fed with turnip. However, these results are only limited to the tested substrates and further production trials are needed^41^. The accumulation of heavy metals (Cd, Pb, Ni, As, Hg) by *T. molitor* was reported with significant correlation to the metal content in the substrates. Although metal concentrations found in the diets were below the legal limit in animal feed^42^. Furthermore, bioaccumulation of arsenic was found in mealworm larvae, while bioaccumulation of Cd and Pb did not occur^43^. Knowledge regarding the effect of diets on the nutritional profile of *T. molitor* is crucial for its safe implementation in food and feed products.

The research reported in this paper focuses on the effect of using agricultural sidestreams as wet feed source on the nutritional composition of *T. molitor*. Besides dry feed, the larvae must also be provided with wet feed. The wet feed source provides the necessary moisture and, furthermore, it also serves as a nutritional supplement for *T.molitor*, increasing the growth rate and maximum weight^44,45^. Based on our data on standard mealworm breeding practices within the Interreg-Valusect project, the total feed of *T. molitor* contains 57 wt% of wet feed. Commonly, fresh vegetables such as carrots are used as a wet feed source^35,36,42,44,46^. Using low-value sidestreams as a wet feed source will provide insect breeding an increased sustainable and economic advantage^17^. The objectives for current study are : (1) study the influence of using biowaste as wet feed on the nutritional composition of *T. molitor*, (2) determine the macro- and micromineral contents of the *T. molitor* larvae bred on mineral-rich biowaste to verify the possibility for mineral-enrichment, and (3) evaluate the safety of these sidestreams in insect breeding for food and feed by analyzing the presence and accumulation of the heavy metals Pb, Cd and Cr. This study will provide more insights in the influence of supplementing biowaste to the diet of *T. molitor* larvae on their nutritional profile and safety.

## Results

### Proximate analysis of wet feed and mealworm larvae

The proximate composition of the wet feeds and mealworm larvae are presented in Table 1.

**Table 1:** Proximate analysis and pH value of wet feeds and proximate analysis of yellow mealworm larvae biomass.

Wet feed composition							
Wet feed	DM^1^	CA^2^	EE^2^	CP^2^	aNDF^2^	NFC^2^	pH
Agar (Control)	2.2 ± 0.0^a^	1.4 ± 0.0^a^	0.0 ± 0.0^a^	0.0 ± 0.1^a^	18.1 ± 2.6^ab^	80.2 ± 1.6^a^	7.3 ± 0.0^a^
Vegetable mix	5.6 ± 0.1^b^	11.9 ± 0.1^b^	2.4 ± 0.3^b^	13.4 ± 0.0^b^	20.9 ± 0.2^ab^	51.4 ± 0.8^b^	5.1 ± 0.0^b^
Potato cuttings	29.9 ± 1.3^c^	2.9 ± 0.1^a^	0.1 ± 0.1^c^	4.9 ± 0.1^c^	13.8 ± 1.0^b^	78.3 ± 1.6^a^	4.7 ± 0.0^c^
Fermented chicory roots	13.4 ± 0.3^d^	47.1 ± 1.6^c^	1.1 ± 0.1^d^	4.4 ± 0.2^d^	15.5 ± 0.1^b^	31.9 ± 1.5^b^	3.7 ± 0.0^d^
Horticultural foliage	7.5 ± 0.2^e^	35.8 ± 0.4^d^	1.2 ± 0.0^e^	15.0 ± 0.2^e^	21.2 ± 0.3^a^	26.8 ± 1.0^c^	8.8 ± 0.0^e^

Mealworm biomass composition							
Wet feed fed	DM^1^	CA^2^	EE^2^	CP^2^	Chitin^2^	NFC^2^	
Agar (Control)	28.3 ± 2.3^a^	4.7 ± 0.6^a^	18.6 ± 1.9^a^	51.4 ± 1.6^a^	7.6 ± 0.5^a^	17.7 ± 2.1^a^	
Vegetable mix	29.4 ± 1.9^ab^	5.0 ± 0.5^a^	19.9 ± 2.9^a^	48.8 ± 2.4^a^	6.9 ± 0.4^ab^	19.4 ± 2.8^a^	
Potato cuttings	35.3 ± 0.7^b^	3.7 ± 0.3^a^	35.5 ± 1.6^b^	40.7 ± 0.5^b^	6.1 ± 0.2^b^	14.0 ± 1.8^b^	
Fermented chicory roots	30.8 ± 1.6^ab^	6.8 ± 0.9^b^	20.0 ± 0.2^a^	48.2 ± 1.0^a^	7.8 ± 0.1^a^	17.2 ± 1.9^ab^	
Horticultural foliage	28.9 ± 2.7^a^	4.7 ± 0.2^a^	19.1 ± 1.5^a^	52.3 ± 0.7^a^	7.7 ± 0.7^a^	16.1 ± 2.4^ab^	

With dry matter content (DM), crude ash content (CA), fat content or ether extract (EE), crude protein content (CP), amylase-treated neutral detergent fiber content (aNDF), non-fiber carbohydrate content (NFC), chitin and pH. Values are reported as mean ± standard deviation (n = 3). ^1^g/100 g fresh matter, ^2^g/100 g dry matter. a–d, values not connected by the same letter are significantly different.

The sidestreams had a higher dry matter content compared to the control wet feed, agar. Vegetable mix and horticultural foliage had a dry matter content below 10%, whereas potato cuttings and fermented chicory roots had a higher dry matter content (13.4 and 29.9 g/100 g fresh matter, FM).

Compared to the control feed (agar), the vegetable mix showed a higher crude ash, fat and protein content, while the non-fiber carbohydrate content was lower and the amylase treated neutral detergent fiber content was similar. Potato cuttings had the highest amount of carbohydrates of all sidestreams and comparable to the carbohydrate content of agar. In general, its proximate composition was most similar to the control feed but supplemented with a small percentage of proteins (4.9%) and crude ash (2.9%)^47,48^. Potatoes have a pH value between 5 and 6, and it was noted that this potato sidestream was more acidic (4.7). Fermented chicory roots were very rich in ash and was the most acidic sidestream of all. As the roots had not been washed, it was expected that most of the ash consisted out of sand (silica). Horticultural foliage was the sole alkaline product compared to the control and other sidestreams. It contained elevated levels of ash and protein compared to the control and a much lower carbohydrate content. The proximate composition was most comparable to fermented chicory roots but with a higher concentration of crude proteins (15.0%) which was comparable to the protein content of the vegetable mix. The statistical analysis of the above data showed that the sidestreams had significant differences in proximate composition and pH.

The addition of vegetable mix or horticultural foliage to the mealworm diet had no influence on the composition of the mealworm larvae biomass compared to the control (Table 1). Adding potato cuttings resulted in the most significant different biomass composition compared to the mealworm larvae fed with control and other wet feed sources. Regarding the protein level of the mealworms, the varied proximate composition of the sidestreams did not affect the protein content of the larvae, except for potato cuttings. Feeding potato cuttings as a moisture souce resulted in doubling the fat content of the larvae, while the contents of protein, chitin and non-fibre carbohydrates decreased. Fermented chicory roots increased the ash content of the mealworm larvae one-and-a-half times.

### Mineral profiles of wet feed and mealworm larvae

The mineral profiles are presented as contents of macrominerals (Table 2) and microminerals (Table 3) for the wet feed and the mealworm larvae biomass.

**Table 2:** Concentration of the macrominerals in the wet feeds and mealworm larvae biomass.

Wet feed macromineral composition					
Wet feed	P	Mg	K	Na	Ca
Agar (Control)	23 ± 1^a^	34 ± 1^a^	3 ± 3^a^	111 ± 0^a^	336 ± 7^a^
Vegetable mix	619 ± 25^b^	199 ± 9^b^	4057 ± 97^b^	88 ± 4^b^	496 ± 17^b^
Potato cuttings	143 ± 3^c^	27 ± 3^a^	1070 ± 17^c^	3 ± 2^c^	31 ± 2^c^
Fermented chicory roots	263 ± 17^d^	185 ± 13^b^	2340 ± 148^d^	187 ± 16^d^	530 ± 38^b^
Horticultural foliage	785 ± 11^e^	925 ± 18^c^	9909 ± 110^e^	33 ± 0^e^	7276 ± 136^d^

Mealworm biomass macromineral composition					
Wet feed fed	P	Mg	K	Na	Ca
Agar (Control)	1095 ± 112^a^	274 ± 32^ab^	1216 ± 131^a^	115 ± 42^ab^	24 ± 4^a^
Vegetable mix	939 ± 38^ab^	297 ± 14^a^	1128 ± 27^ab^	105 ± 60^ab^	28 ± 3^a^
Potato cuttings	690 ± 30^b^	165 ± 13^c^	891 ± 45^b^	96 ± 11^ab^	2 ± 0^b^
Fermented chicory roots	971 ± 32^a^	244 ± 9^abc^	1219 ± 33^ab^	175 ± 5^a^	79 ± 5^c^
Horticultural foliage	1065 ± 14^a^	222 ± 14^bc^	1206 ± 20^a^	63 ± 2^b^	66 ± 7^c^

Values are reported as mean ± standard deviation (n = 3). Concentrations are expressed as mg/100 g DM. a–e, values not connected by the same letter are significantly different.

**Table 3:** Concentration of the microminerals in tested wet feed and mealworm larvae biomass.

Wet feed micromineral composition				
Wet feed	Zn	Cu	Fe	Mn
Agar (Control)	2.03 ± 0.05^a^	0.73 ± 0.04^a^	5.72 ± 0.14^a^	11.92 ± 0.60^a^
Vegetable mix	2.79 ± 0.08^b^	0.74 ± 0.03^a^	7.99 ± 0.28^a^	3.46 ± 0.15^b^
Potato cuttings	0.67 ± 0.03^c^	0.36 ± 0.03^b^	3.68 ± 0.10^a^	1.32 ± 0.17^c^
Fermented chicory roots	4.72 ± 0.49^d^	2.15 ± 0.13^c^	521.80 ± 20.63^b^	13.22 ± 0.80^a^
Horticultural foliage	3.70 ± 0.02^e^	0.62 ± 0.02^a^	12.88 ± 0.36^a^	30.95 ± 1.54^d^

Mealworm biomass micromineral composition				
Wet feed fed	Zn	Cu	Fe	Mn
Agar (Control)	13.60 ± 1.20^a^	2.23 ± 0.16^a^	5.20 ± 2.50^a^	1.37 ± 0.39^a^
Vegetable mix	15.78 ± 3.18^a^	2.01 ± 0.07^ab^	5.73 ± 0.68^a^	1.24 ± 0.05^a^
Potato cuttings	12.30 ± 1.44^a^	2.11 ± 0.27^ab^	4.58 ± 0.38^a^	1.58 ± 0.07^ab^
Fermented chicory roots	12.80 ± 0.43^a^	2.37 ± 0.06^a^	45.19 ± 3.06^b^	2.31 ± 0.07^b^
Horticultural foliage	13.82 ± 0.30^a^	1.76 ± 0.07^b^	4.56 ± 0.20^a^	1.58 ± 0.04^ab^

Values are reported as mean ± standard deviation (n = 3). Concentrations are expressed as mg/100 g DM. a–e, values not denoted by the same letter are significantly different.

In general, the agricultural sidestreams were richer in macrominerals compared to the control, except for potato cuttings which had lower contents of Mg, Na and Ca. The concentration of K was abundant in all sidestreams compared to the control. Agar contained 3 mg/100 g DM of K while the concentration of K in the sidestreams ranged from 1070 to 9909 mg/100 g DM. Vegetable mix contained significantly higher levels of macrominerals than the control, except for the Na content which was significantly lower (88 vs 111 mg/100 g DM). Potato cuttings contained the lowest concentration of macrominerals of all sidestreams. The macrominerals in potato cuttings were significantly lower than other sidestreams and control. Except for the Mg content which was comparable with the control. While fermented chicory roots did not hold the highest concentrations of macrominerals, the ash content of this sidestream was the highest of all sidestreams. This was probably due to the fact that they were unwashed and potentially held high concentrations of silica (sand). The amount of Na and Ca were comparable to the amounts in vegetable mix. Fermented chicory roots contained the highest concentration Na of all sidestreams. Horticultural foliage had the highest macro mineral concentrations of all wet feed, except for Na. The concentration of K (9909 mg/100 g DM) was three thousand times higher than the control (3 mg/100 g DM) and two-and-a-half times higher than the concentration of K in vegetable mix (4057 mg/100 g DM). The content of Ca was highest of all sidestreams (7276 mg/100 g DM), twenty times higher than the control (336 mg/100 g DM) and fourteen times higher than the concentration of Ca in fermented chicory roots or vegetable mix (530 and 496 mg/100 g DM).

There was no significant difference in macromineral composition of the mealworms grown on the vegetable mix or the control wed feed, even though there were significant differences in feed macromineral composition (Table 2).

The larvae fed with potato cuttings showed significantly lower concentrations of all macrominerals compared to the control, except Na, which was comparable. Additionally, when compared to the other sidestreams, feeding potato cuttings decreased the larval macromineral contents the most. This was in line with the lower ash content that was observed in the proximate composition of the mealworms. However, while P and K were significantly higher in this wet feed compared to the other sidestreams and control, the larval composition did not reflect this. The low concentration of Ca and Mg found in the mealworm biomass could be related to the low concentration of Ca and Mg present in the wet feed itself.

Feeding fermented chicory roots and horticultural foliage lead to a substantial increase in Ca compared to the control. The horticultural foliage contained the highest levels of P, Mg, K and Ca of all wet feeds, but this was not reflected in the mealworm biomass. The Na content in these larvae was the lowest of all, while the Na concentration in the horticultural foliage was higher compared to the potato cuttings. An increase was found in the Ca content (66 mg/100 g DM) of the larvae, but the concentration of Ca was not as high as in mealworm biomass from the fermented chicory roots experiment (79 mg/100 g DM), even though the horticultural foliage had a fourteen times higher concentration of Ca compared to fermented chicory roots.

When looking at the content of the microminerals (Table 3) of the wet feed, the mineral profile of the vegetable mix was similar to the control, except for a significantly lower concentration of Mn. The potato cuttings contained lower concentrations of all analyzed microminerals compared to the control and the other sidestreams. The fermented chicory roots contained almost 100 times more Fe, four times more Cu and twice as much Zn than the control, while the Mn content was comparable between them. The horticultural foliage contained significantly more Zn and Mn than the control.

No significant differences were found between the micromineral content of the larvae fed on the control, vegetable mix and potato cuttings wet feed. The Fe and Mn content of fermented chicory roots fed larvae was, however, significantly different compared to the control fed mealworms. The increase in Fe could be linked with a hundred times higher concentration of the micromineral in the wet feed itself. However, while the Mn concentration found in the fermented chicory roots and the control- were not significantly different, an increase was found in the Mn content of the larvae fed with fermented chicory roots. Notice also how the higher concentration of Mn (3×) in the horticultural foliage wet feed compared to the control was not noticeable in the mealworm biomass composition. The only difference between the control and horticultural foliage was the Cu content, which was lower for the foliage.

### Heavy metal concentrations in substrates and mealworm larvae

The concentrations of heavy metals found in the substrates are presented in Table 4. The European maximum levels for Pb, Cd and Cr in complete animal feed were recalculated to mg/100 g DM and are added to Table 4 to compare the concentrations found in the side streams^47^.

**Table 4:** Concentration of heavy metals in tested substrates and mealworm larvae biomass.

Substrate heavy metal content			
Substrates	Pb	Cd	Cr
Agar (Control)	0.000 ± 0.000^a^	0.000 ± 0.000^a^	0.023 ± 0.000^a^
Vegetable mix	0.000 ± 0.000^a^	0.000 ± 0.000^a^	0.007 ± 0.000^a^
Potato cuttings	0.000 ± 0.000^a^	0.000 ± 0.000^a^	0.004 ± 0.001^a^
Fermented chicory roots	0.041 ± 0.006^b^	0.000 ± 0.000^a^	0.135 ± 0.011^b^
Horticultural foliage	0.002 ± 0.001^a^	0.000 ± 0.000^a^	0.021 ± 0.010^a^
Max. EU level for animal feed	0.440	0.044	–

Mealworm biomass heavy metal content			
Mealworm larvae	Pb	Cd	Cr
Agar (Control)	0.041 ± 0.024^a^	0.000 ± 0.000^a^	0.040 ± 0.025^a^
Vegetable mix	0.010 ± 0.001^c^	0.000 ± 0.000^a^	0.058 ± 0.011^a^
Potato cuttings	0.017 ± 0.008^ac^	0.000 ± 0.000^a^	0.056 ± 0.011^a^
Fermented chicory roots	0.109 ± 0.014^b^	0.000 ± 0.000^a^	0.154 ± 0.038^b^
Horticultural foliage	0.050 ± 0.007^a^	0.000 ± 0.000^a^	0.041 ± 0.001^a^

Values are reported as mean ± standard deviation (n = 3). Concentrations are expressed as mg/100 g DM. a–c, values not denoted by the same letter are significantly different.

Pb was not detected in the control wet feed, vegetable mix or potato cuttings, while horticultural foliage contained 0.002 mg Pb/100 g DM and fermented chicory roots contained the highest concentration of 0.041 mg Pb/100 g DM. The concentration of Cr in the control feed and horticultural foliage were comparable (0.023 and 0.021 mg/100 g DM), while the vegetable mix and potato cuttings had a lower concentration of Cr (0.004 and 0.007 mg/100 g DM). The concentration of Cr was significantly higher in fermented chicory roots (0.135 mg/100 g DM) compared to the other substrates and six times higher than the control feed. Cd was not detected in the control feed nor in any used sidestream.

Significantly higher levels of Pb and Cr were found in the larvae fed with fermented chicory roots. Cd was not detected in any of the mealworm larvae.

### Fatty acid profile of mealworm larvae

A qualitative analysis of the fatty acids in the crude fat was done to check if fatty acid profiles of the mealworm larvae would be influenced by the different compositions of the sidestreams they were fed with. The distribution of these fatty acids is shown in Table 5. Fatty acids are tabled by their trivial name and their molecular structure (expressed as “Cx:y” in which x equals the number of carbons and y equals the amount of unsaturated bounds).

**Table 5:** Fatty acid profiles of mealworm larvae reared on feed supplemented with sidestreams.

Fatty Acid	Control	Vegetable mix	Potato cuttings	Fermented chicory roots	Horticultural foliage
C14:0 (myristic acid)	1.65 ± 0.18^a^	2.29 ± 0.08^c^	2.95 ± 0.06^d^	1.95 ± 0.05^ab^	2.06 ± 0.07^bc^
C15:0 (pentadecanoic acid)	0.18 ± 0.03^a^	0.18 ± 0.01^a^	0.13 ± 0.02^c^	0.20 ± 0.01^a^	0.25 ± 0.01^b^
C16:0 (palmitic acid)	17.96 ± 2.31^a^	18.44 ± 0.63^a^	22.73 ± 0.14^b^	19.91 ± 0.08^a^	19.33 ± 0.91^a^
C16:1 (palmitoleic acid)	0.48 ± 0.08^a^	0.57 ± 0.04^a^	1.34 ± 0.07^b^	0.67 ± 0.02^a^	0.50 ± 0.04^a^
C16:2 (Hexadecadienoic acid)	0.31 ± 0.09^a^	0.34 ± 0.01^a^	0.20 ± 0.01^b^	0.25 ± 0.01^ab^	0.34 ± 0.03^a^
C17:0 (heptadecanoic acid)	0.22 ± 0.09^a^	0.21 ± 0.01^a^	0.10 ± 0.00^b^	0.21 ± 0.02^a^	0.26 ± 0.01^a^
C18:0 (stearic acid)	2.78 ± 0.62^a^	2.55 ± 0.12^a^	2.25 ± 1.95^a^	2.67 ± 0.12^a^	2.87 ± 0.04^a^
C18:1 (oleic acid)	27.06 ± 2.54^a^	29.92 ± 0.87^a^	43.19 ± 2.86^b^	32.91 ± 0.59^a^	30.52 ± 0.69^a^
C18:2 (linoleic acid)	39.89 ± 5.90^a^	43.80 ± 1.10^a^	26.29 ± 1.16^b^	39.83 ± 0.69^a^	42.14 ± 1.55^a^
C18:3 (linolenic acid)	1.21 ± 0.20^a^	1.60 ± 0.17^b^	0.76 ± 0.07^c^	1.28 ± 0.04^a^	1.62 ± 0.17^b^
C20:0 (arachidic acid)	0.11 ± 0.01^a^	0.10 ± 0.01^ab^	0.08 ± 0.01^b^	0.10 ± 0.01^ab^	0.12 ± 0.02^a^
SFA	22.91 ± 0.98^a^	23.77 ± 0.93^a^	28.23 ± 1.47^b^	25.05 ± 0.53^a^	24.89 ± 1.03^a^
MUFA	27.53 ± 1.62^a^	30.50 ± 1.61^a^	44.53 ± 2.55^b^	33.59 ± 1.16^a^	31.02 ± 1.65^a^
PUFA	41.41 ± 2.49^a^	45.74 ± 2.06^a^	27.24 ± 2.60^b^	41.37 ± 1.60^a^	44.09 ± 2.21^a^

Values are reported as mean ± standard deviation (n = 3) and expressed % fatty acid (group) of 100% crude fat. Fatty acids are expressed as “Cx:y” in which x equals the number of carbons and y equals the amount of unsaturated bounds. Fatty acids are grouped as saturated fatty acids (SFA), mono-unsaturated fatty acids (MUFA) and poly-unsaturated fatty acids (PUFA) a–c, values not denoted by the same letter are significantly different.

A remarkable change was observed of the fatty acid profile of mealworms fed with potato cuttings. They contained significantly higher concentrations of myristic acid (C14:0), palmitic acid (C16:0), palmitoleic acid (C16:1) and oleic acid (C18:1). The concentrations of pentadecanoic acid (C15:0), linoleic acid (C18:2) and linolenic acid (C18:3) were significantly lower compared to the other mealworms. The ratio of C18:1 to C18:2 was reversed for potato cuttings compared to the other fatty acid profiles. Mealworms fed with horticultural foliage contained a higher amount of pentadecanoic acid (C15:0) compared to the other wet feed fed mealworms.

Fatty acids were grouped as Saturated Fatty Acids (SFA), Mono-Unsaturated Fatty Acids (MUFA) and Poly-Unsaturated Fatty Acids (PUFA). Table 5 shows the concentrations of these fatty acid groups. In general, the fatty acid profile of mealworm fed with potato cuttings differed significantly from the control group and other sidestreams. For each fatty acid group, mealworms fed with potato cuttings differed significantly from all the others. They contained more SFA’s and MUFA’s and less PUFA’s.

## Discussion

There were no significant differences between the survival rates and the total harvested weights of larvae that were bred on the different substrates. The overall mean survival rate was 90% and the overall mean total harvested weight was 974 g. The side streams were succesfully processed by the mealworms as a wet feed source. The wet feed of mealworms exists out of more than half the total given feed mass (dry + wet). Replacing fresh vegetables as the traditional wet feed by agricultural biowaste sidestreams has an economic and ecological advantage for mealworm breeding.

Table 1 shows that mealworm larvae grown on the control diet had a proximate biomass composition of 72% moisture and on a dry matter basis 5% ash, 19% lipid, 51% protein, 8% chitin and 18% non-fibre carbohydrate which was comparable to the values reported in the literature^48,49^. However, other compositions can be found in literature, often depending on the analytical methods that were used. For example, we determined the crude protein content using the Kjeldahl method with an N to P factor of 5.33, while other researchers calculate with a more widely used factor of 6.25, for meat and feed samples^50,51^.

The addition of potato cuttings, a wet feed high in carbohydrates, to the diet resulted in doubling the fat content of the mealworms. It was expected that the carbohydrate content of potatoes mainly consisted out of starch whereas agar contained sugars (polysaccharides)^47,48^. This finding was contradictory to another study that found a decrease in the fat content when mealworms were reared on a diet supplemented with potato steam pealings which was low in protein (10.7%) and high in starch (49.8%)^36^. When olive pomace was added to the diet, mealworms reflected the wet feed compositions in their protein and carbohydrate content, while the fat content remained unchanged^35^. Contradictory, other studies reported that the protein content of larvae bred on sidestreams did fundamentally change, as well as the fat content^22,37^.

Fermented chicory roots significantly increased the ash content of the mealworm larvae (Table 1). Research on the influence of sidestreams on the ash and mineral content of mealworm larvae is limited. Most of the research that is published on feeding by-products focusses on the fat and protein content of the larvae and ash content is not analysed^21,35,36,38,39^. However, when ash content was analyzed for larvae that were fed with by-products, an increase was found. For example, feeding garden waste to mealworms increased their ash content from 3.01 to 5.30%^37^ and adding watermelon waste to the diet increased the ash content from 1.87 to 4.40%^52^.

While all wet feed sources differed significantly from each other in proximate composition (Table 1), less significant differences were found between the biomass compositions of the mealworm larvae fed with the respective wet feeds. Only mealworm larvae fed with potato cuttings or fermented chicory roots had undergone considerable changes. A possible explanation for this outcome could be the fact that besides the chicory roots, also the potato cuttings were partly fermented (pH 4.7, Table 1) making the starch/carbohydrates more digestible/available for the mealworm larvae. How mealworm larvae synthesise lipids out of nutrients like carbohydrates is of great interest and future research should be performed to fully understand this. A previous study on the influence of wet feed pH on the growth of mealworm larvae concluded that there were no significant differences when providing wet feed agar cubes in the range from pH 3 to 9. Indicating that fermented wet feed could be provided for mealworm breeding^53^. Similar to Coudron et al.^53^ agar cubes were used in the provided wet feed in the control experiment as they are poor in minerals and poor in nutrients. In their research, the effect of improving digestibility or bioavailability of a wet feed source with a more diverse nutrient profile like vegetables or potatoes was not studied. Further research on the influence of fermentation of wet feed sources for mealworm larvae is necessary to study this theory in-depth.

The mineral profiles of the mealworm biomass of the control group (Tables 2 and 3) found in this study was comparable with the ranges of macro- and microminerals found in the literature^48,54,55^. Providing mealworms fermented chicory roots as a wet feed source had the biggest increase in their mineral contents. While vegetable mix and horticultural foliage were more abundant in most macro- and microminerals (Tables 2 and 3), they did not affect the mineral contents in the mealworm biomass as fermented chicory roots did. A possible explanation could be that nutrients were less bio-available in the alkaline horticultural foliage compared to the more acidic other wet feeds (Table 1). Previous study that fed yellow mealworm larvae with fermented straw found that they seemed to develop well on this sidestream and it was shown that pretreatment of the substrate by fermentation induced the uptake of nutrients^56^. Providing fermented chicory roots elevated the contents of Ca, Fe and Mn in the mealworm biomass. Although this sidestream also held higher concentrations of other minerals (P, Mg, K, Na, Zn and Cu), these minerals did not increase significantly in the mealworm biomass compared to the control indicating that the mineral uptake was selective. Increasing these mineral contents in mealworm biomass can be nutritionally beneficial for food and feed applications. Calcium is an essential mineral, playing vital roles in neuromuscular function and many enzyme-mediated processes like blood clotting, bone and tooth formation^57,58^. Iron deficiency is of widespread concern in developing countries and children, women and the elderly often receive insufficient amounts of Fe from their diet^54^. Although Mn is an essential element in the human diet and has a central role for the function of several enzymes, overexposure can be toxic. The higher level of Mn in the mealworms fed with fermented chicory roots was not alarming and comparable to chicken meat^59^.

The heavy metal concentrations found in the side streams were below the European standards for complete animal feed. The heavy metal analysis of the mealworm larvae showed significantly higher levels of Pb and Cr in the ones fed with fermented chicory roots compared to the control and other substrates (Table 4). Chicory roots are grown in soil and known for their uptake of heavy metals^60^, while other sidestreams were derived from controlled human food production. Mealworms fed with fermented chicory roots also contained higher levels of Pb and Cr (Table 4). The bioaccumulation factors (BAF) calculated were 2.66 for Pb and 1.14 for Cr, thus greater than 1. This indicated the ability to accumulate heavy metals by *T. molitor*. For Pb, the European Union prescribes a maximum level of 0.10 mg Pb per kg fresh meat for human consumption^61^. When evaluating our experimental data, a maximum concentration of Pb was found in fermented chicory roots mealworms with a value of 0.11 mg/100 g DM. Recalculating this value with a 30.8% dry matter content for these mealworms, a value of 0.034 mg Pb/kg fresh matter was found, which was below the maximum level of 0.10 mg/kg. There are no maximum levels for Cr in European food legislation. Cr is commonly present in the environment, food and food supplements and is known to be an essential nutrient for the human body, in small amounts^62–64^. These analyses (Table 4) show the possibility of accumulating heavy metals in the larvae of *T. molitor*, when present in the diet. However, the heavy metal levels found in the mealworm biomass in this study are considered safe for human consumption. Regular close monitoring is advised when using sidestreams that can contain heavy metals as wet feed source for *T. molitor*.

The most abundant fatty acids in the biomass of all *T. molitor* larvae were palmitic (C16:0), oleic (C18:1) and linoleic acid (C18:2) (Table 5), which is comparable with findings in previous studies on the fatty acid profile of *T. molitor*^36,46,50,65^. The fatty acid profile of *T. molitor* is commonly known to exist out of five main components; oleic (C18:1), palmitic (C16:0), linoleic (C18:2), myristic (C14:0) and stearic acid (C18:0). Oleic acid is reported as the most abundantly present fatty acid in mealworm larvae (30–60%), followed by palmitic acid and linoleic acid^22,35,38,39^. Previous research has shown that this fatty acid profile was affected by the diet given to the mealworm larvae, but differences did not follow the same trends as the diets^38^. The ratio of C18:1–C18:2 was reversed for potato cuttings compared to the other fatty acid profiles. Similar results were found on this change of the fatty acid profile of mealworms fed with potato steam peelings^36^. These findings indicate that while it is possible to influence the fatty acid profile of yellow mealworm oil, it remains an abundant source of unsaturated fatty acids.

## Conclusions

This study aimed to evaluate the impact of using four different biowaste streams from agro-industrial by-products as wet feed on the composition of *T. molitor*. Based on the determination of the nutritional profile of the larvae, the effects were assessed. The results showed that the by-products were successfully transformed into protein-rich biomass (40.7–52.3% protein content) which can be used as a source for food and feed. Moreover, the study revealed that using the by-products as wet feed had an effect on the nutritional profile of the *T. molitor* biomass. Specifically, providing the larvae with a high concentration of carbohydrates (i.e. potato cuttings) increased their fat content and changed the fatty acid profile: lower levels of poly-unsaturated fatty acids and higher levels of saturated and mono-unsaturated fatty acids, but the concentration of unsaturated fatty acids (mono + poly) remained dominant. The study also found that *T. molitor* selectively accumulated Ca, Fe and Mn from acidic mineral rich sidestreams. The bioavailability of the minerals seemed to play an important role and further research is necessary to fully understand this. Heavy metals present in the sidestreams could accumulate in *T. molitor*. However, the final concentrations of Pb, Cd and Cr in the larvae biomass were below the allowable limits which makes it safe to use these sidestreams as a wet feed source.

## Methods

### Rearing of Tenebrio molitor

The yellow mealworm larvae were reared by Radius of the Thomas More University of Applied Sciences (Geel, Belgium) and Inagro (Rumbeke-Beitem, Belgium) at a temperature of 27 °C and a relative humidity of 60%. A density of 4.17 mealworms/cm^2^ (10.000 mealworms) was applied in rearing crates of 60 cm × 40 cm. Larvae were given 2.1 kg wheat bran as dry feed per rearing crate at the start and were subsequently supplemented on demand. Agar cubes were used as a control wet feed treatment. From week 4 on, the sidestreams (also the moisture source) were given ad libitum as wet feed instead of agar. The dry matter percentage of each sidestream was determined in advance and was taken into account, providing all insects of the different treatments the same amount of moisture. The feed was distributed evenly throughout the rearing crates. The larvae were harvested when the first pupae appeared in the treatments. Larvae were harvested using a mechanical vibrating sieve of 2 mm. With exception for the experiment with potato cuttings. Larger parts of potato cuttings that were dried out were additionally separated by letting the larvae crawl through this sieve and collect them in a metal pan. Total harvest weight was determined by weighing this total harvest. Survival rate was calculated by dividing total harvest weight by larval weight. Larval weight was determined by picking a minimum of 100 larvae and divide their total weight by the amount. Prior to analysis, the harvested larvae were starved for 24 h to empty their gut. Finally, the larvae were sieved again to separate them from their residue. They were euthanized by freezing and stored at − 18 °C until analysis.

### Biowaste as insect feed

**Figure 1:**
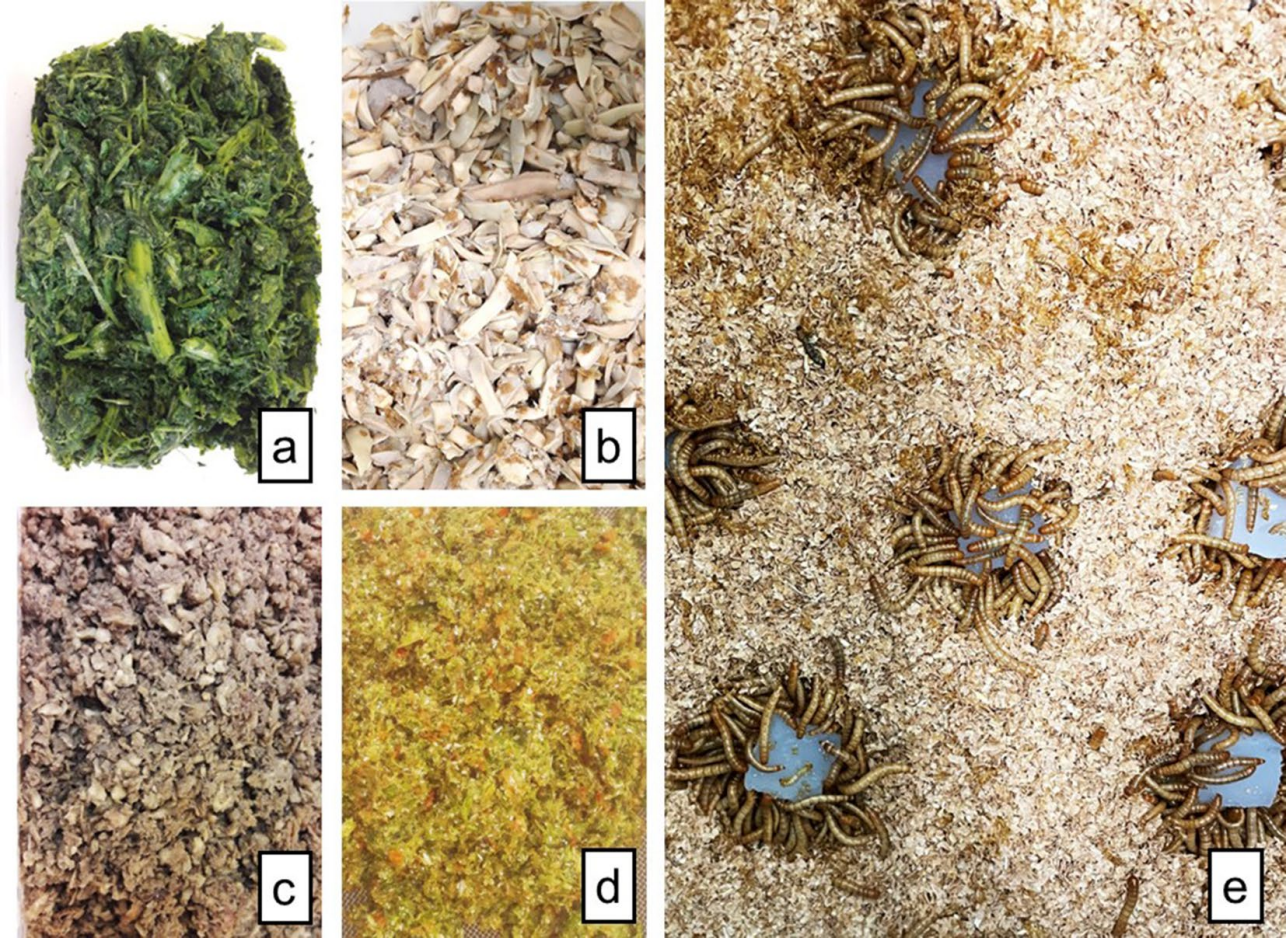
Agricultural sidestreams as wet feed for yellow mealworms; (**a**) horticultural foliage from cucumber cultivation, (**b**) potato cuttings, (**c**) forced chicory roots, (**d**) unsold vegetables from auction and (**e**) agar cubes as control.

As a dry feed, wheat bran (Molens Joye, Belgium) was given. The wheat bran was sieved in advance, with a particle size below 2 mm. Besides dry feed, mealworm larvae must also be provided with a wet feed which holds the necessary moisture and supplemental minerals for *T. molitor*. Wet feed exists out of more than half the total given feed (dry + wet). Agar (Brouwland, Belgium, 25 g/L) was provided as control wet feed in our experiment^45^. Four nutritionally varying, agricultural sidestreams were tested as a wet feed for *T. molitor* larvae as represented in Fig. 1. These sidestreams included (a) foliage from cucumber cultivation (Inagro, BE), (b) potato cuttings (Duynie, BE), (c) fermented chicory roots (Inagro, BE) and (d) unsold food from fruit and vegetable auction (Belorta, BE). Sidestreams were shredded/shopped into pieces to make them suitable as wet feed for the mealworms.

### Analytical methods

The composition of feed and mealworm larvae were determined in triplicate (n = 3). Evaluations were performed on proximate analysis, mineral profile, heavy metal content and fatty acid profile. A homogeneous sample of 250 g was taken from the harvested and starved larvae, dried at 60 °C to constant weight, grinded (IKA, Tube mill 100) and sieved through a 1 mm screen. Dried samples were stored air-tight in a dark container.

#### Dry matter content

Dry matter content (DM) was determined by drying the sample in an oven at 105 °C for 24 h (Memmert, UF110). Dry matter percentage was calculated based on the weight loss of the sample.

$$\% {\text{DM }} = {\text{ m}}_{{{\text{dried sample}},{ 1}0{5}\;^\circ {\text{C}}}} /{\text{ m}}_{{{\text{sample}}}} \times { 1}00$$

#### Crude ash content

Crude ash content (CA) was determined from the weight loss by incineration in a muffle furnace (Nabertherm, L9/11/SKM) at 550 °C for 4 h.

$$\% {\text{Ash }} = {\text{ m}}_{{\text{incinerated sample}}} /{\text{m}}_{{{\text{dried sample}},{ 1}0{5}\;^\circ {\text{C}}}} \times { 1}00$$

#### Crude fat content or ether extraction

Crude fat content or ether extraction (EE) was performed with petroleum ether (BP 40–60 °C) using soxhlet equipment. About 10 g of sample was placed in an extraction thimble and covered with ceramic wool to prevent sample loss. The sample was extracted overnight with 150 mL of petroleum ether. The extract was cooled down and the organic solvent was removed and recovered via rotary evaporation (Büchi, R-300) at 300 mbar and 50 °C. The crude lipid or ether extract was cooled down and weighted on an analytical balance.

#### Crude protein content

Crude protein content (CP) was determined by analyzing the nitrogen present in the samples using the Kjeldahl method BN EN ISO 5983-1 (2005). Appropriate N to P factors were used to calculate the protein content. For standard dry feed (wheat bran), the general factor of 6.25 was used. For the sidestreams a factor of 4.23^66^ was applied, except for the Vegetable Mix for which a factor of 4.39 was used^67^. Crude protein content of the larvae was calculated with a N to P factor of 5.33^51^.

#### Neutral detergent fibre

The fibre content included the determination of Neutral Detergent Fibre (NDF) based on the extraction protocols of Gerhardt (Manual Fibre bag analysis, Gerhardt, Germany) and the Van Soest method^68^. For NDF determination, a weighted sample of 1 g was placed in a dedicated fibre bag (Gerhardt, ADF/NDF bag) with glass spacer. The fibre bag filled with sample was first defatted in petroleum ether (BP 40–60 °C) and dried at room temperature. Defatted sample was extracted in a neutral detergent fibre solution with heat-stable alpha-amylase at boiling temperature for 1.5 h. Subsequently, the sample was rinsed three times with boiling, demineralised water and dried overnight at 105 °C. The weight of the dried fibre bag with fibre residue was determined on an analytical balance (Sartorius, P224-1S), followed by incineration at 550 °C for 4 h in a muffle furnace (Nabertherm, L9/11/SKM). The weight of the ashes was determined again and fibre content was calculated based on the weight loss between dried and incinerated sample.

#### Chitin content

To determine the chitin content of the larvae, an adapted protocol was used based on analysis of crude fibre content following the Van Soest method^68^. A weighted sample of 1 g was placed in a dedicated fibre bag (Gerhardt, CF bag) and glass spacer. The fibre bag was filled with sample, defatted in petroleum ether (BP 40–60 °C) and dried in atmosphere. The defatted sample was first extracted in an acidic solution of 0.13 M sulphuric acid for 30 min at boiling temperature. The extracted fibre bag with sample was rinsed three times with boiling, demineralised water and subsequently extracted in solution of 0.23 M potassium hydroxide for 2 h. The extracted fibre bag with the sample was again rinsed three times with boiling, demineralised water and dried overnight at 105 °C. The dried bag with fibre residue was weighted on an analytical balance and incinerated at 550 °C for 4 h in a muffle furnace. Ashes were weighted and fibre content was calculated based on the weight loss from the incinerated sample.

#### Total carbohydrate content

Total carbohydrate content was determined based on calculations. The non fibre carbohydrates (NFC) concentration is calculated using the NDF analysis for feed and using the chitin analysis for the insects.

1 $$\% {\text{NFC}}\; \, = \;{ 1}00 - \left( {\% {\text{CP }} + \, \% {\text{NDF}}/{\text{chitin }}\; + \; \, \% {\text{EE}}\; \, + \; \, \% {\text{CA}}} \right)$$

#### pH measurement

pH of the substrates was determined according to NBN EN 15933 after extraction in demineralised water (1:5 V/V).

#### Mineral profile

Samples were prepared as described by Broeckx et al.^69^. The mineral profile was determined using ICP-OES (Optima 4300™ DV ICP-OES, Perkin Elmer, Massachusetts, USA).

#### Heavy metal content

The heavy metal content of Cd, Cr and Pb was analysed with graphite furnace atomic absorption (AAS) (Thermo Scientific, ICE 3000 series with GFS Furnace Autosampler). About 200 mg of sample was digested in acidic solution of HNO_3_/HCl (1:3 V/V) using a microwave (CEM, MARS 5). Microwave digestion was performed at 190 °C for 25 min with 600 W power. The extract was diluted with ultrapure water.

#### Fatty acid content

Fatty acids were determined by GC–MS (Agilent Technologies, 7820A GC system with 5977 E MSD detector). Fatty acid methyl esters (FAMEs) were prepared from the ether extracts after esterification in a methanolic KOH solution with addition of 20% BF_3_/MeOH solution according to the method of Joseph and Ackmann^70^. Fatty acids were identified by comparing their retention time with a 37 FAME-mix standard (Chem-lab) or by comparing their MS-spectrum with online libraries such as NIST-database. A qualitative analysis was performed by calculation of the area-percentage of a peak relative to the total peak-area of the chromatogram.

### Data analysis

The data were analysed using the JMP Pro 15.1.1 software package from SAS (Buckinghamshire, UK). Evaluation was done using one-way ANOVA at a significance level of 0.05, with Tukey HSD as post hoc test.

The bioaccumulation factor (BAF) was calculated by dividing the concentration of the heavy metal in the mealworm larval biomass (DM) by the concentration in the wet feed (DM)^43^. A BAF greater than 1 implies bioaccumulation of the heavy metal from the wet feed into the larvae.

## Data Availability

The datasets generated during and/or analysed during the current study are available from the corresponding author on reasonable request.
